# Euglycemic Diabetic Ketoacidosis in a Sedated Patient after Coronary Artery Bypass Grafting: A Case Report and Literature Review

**DOI:** 10.1155/2021/2086520

**Published:** 2021-11-18

**Authors:** Mohamad S. Alabdaljabar, Khaled M. Abdullah, Ali Almasood, Syed Salman Ali, Abdullah Ashmeg

**Affiliations:** ^1^College of Medicine, Alfaisal University, Riyadh, Saudi Arabia; ^2^Cardiac Center, Specialized Medical Center, Riyadh, Saudi Arabia; ^3^Endocrinology Department, Specialized Medical Center, Riyadh, Saudi Arabia

## Abstract

Euglycemic diabetic ketoacidosis (EDKA) is a rare and serious adverse effect of sodium-glucose cotransporter 2 inhibitors (SGLT-2i). The diagnosis is challenging due to the rarity, nonspecific symptoms, and absence of the alarmingly high blood glucose levels, and thus, it could be initially missed resulting in delayed treatment. This is particularly important for sedated patients, as the absence of typical clinical signs and symptoms can obscure the diagnosis. We present the case of a patient with type 2 diabetes mellitus on empagliflozin who developed EDKA while sedated after coronary artery bypass grafting (CABG) despite stopping the medication 24 hours prior to surgery. We also summarize the current literature on EDKA after CABG. Physicians must be aware and maintain a high index of suspicion for the illness, especially in patients with T2DM taking SGLT-2i and undergoing a major operation such as CABG. Emergent treatment and multidisciplinary follow-up are needed to improve patient outcomes and mitigate complications. Physicians should also consider identifying the optimal time to discontinue SGLT-2i before major surgeries and possible ketone studies in such patients, especially those sedated following the surgery.

## 1. Introduction

Diabetic ketoacidosis (DKA) is one of the most serious complications of diabetes mellitus (DM). It is characterized by hyperglycemia (>250 mg/dL), metabolic acidosis (pH < 7.3 and serum bicarbonate <18 mmol/L), and ketosis [1]. This complication typically develops in patients with type 1 DM and, to a lesser extent, type 2 DM (T2DM). It is commonly associated with stress, such as that associated with major surgery or infection. Rarely, these patients could present with a relatively lower blood glucose (BG) level (<250 mg/dL), which is referred to as “euglycemic” DKA (EDKA) [1]. Although it is rare, this entity has been described in the literature. In fact, it was reported that approximately 2.6%–3.2% of all DKA admissions are euglycemic [2].

Among different associated factors, sodium-glucose cotransporter-2 inhibitors (SGLT-2i) (i.e., gliflozins) are believed to be linked to EDKA, with rates ranging from 0.16 to 0.76 events per 1000 patient-years in patients with T2DM [2, 3]. This rare variant of DKA poses a challenging diagnosis for physicians as patients with normal or near-normal BG levels may be initially overlooked resulting in delayed diagnosis and treatment, which can be fatal. EDKA related to SGLT-2i is important to consider in diabetic patients with cardiovascular diseases (CVD). The use of SGLT-2i has become more common in this population after data suggesting a mortality benefit [4]. T2DM patients with cardiac diseases taking gliflozins are prone to experience many stressors, especially when complicated with an acute CVD event (e.g., myocardial infarction, heart failure exacerbation, and cardiac surgeries), which can further increase their chances of getting gliflozin-induced EDKA. In the same context, we present a case of a T2DM patient on SGLT-2i who developed EDKA, while sedated, after coronary artery bypass surgery (CABG). Moreover, we sought to conduct a literature search on EDKA after CABG to shed the light and attract the attention of the medical community to this rising issue.

## 2. Case Presentation

A 52-year-old male, with the past medical history significant for T2DM, hypertension, dyslipidemia, and ischemic heart disease (IHD), presented to the cardiology clinic complaining of recurrent chest pain. He has had chest pain for a long period of time, which was getting more severe one week prior to hospital admission. He denied any history of smoking, illicit drug use, or alcohol consumption. At that time, his physical examination was unremarkable. He was on vildagliptin-metformin (50/1000 mg), empagliflozin (10 mg), pioglitazone (30 mg), irbesartan-hydrochlorothiazide (300/12.5 mg), atorvastatin (20 mg), and aspirin; however, he was not compliant, which was associated with poorly controlled blood pressure and glucose levels. His laboratory results (Table 1) were within normal limits, except for abnormal HbA1c (10.24%) and lipid profile. His ECG showed sinus tachycardia with no acute ischemic changes. Echocardiography showed an ejection fraction (EF) of >50% with no wall motion abnormalities. CT coronaries showed 70% stenosis at the left main coronary artery. Due to these findings, the patient was advised to undergo diagnostic coronary angiography which revealed triple vessel disease. After thorough discussion between the patient, cardiology, and cardiac surgery physicians, the decision was made to operate on the patient with coronary artery bypass grafting (CABG). Owing to his critical lesions, the patient was admitted and closely monitored until the day of surgery. At that time, he was started on insulin sliding scale. Additionally, metformin-vildagliptin was held before coronary angiography, while the other medications, including empagliflozin, were continued until 24 hours prior to the surgery. His laboratory results were normal (Table 1), except for high fasting glucose. He was fasting for a total of 8 hours before the surgery.

The patient underwent grafting of the diseased vessels. The patient tolerated the surgery well, came off the cardiopulmonary bypass without difficulties, and did not need inotropic support. He was shifted to the ICU in a stable condition. On the same day, the patient was sedated on mechanical ventilation. Initially, he was clinically and vitally stable on minimal noradrenaline dose with no oozing from drain sites. Six hours after leaving the OR, his arterial blood gases (ABG) showed anion gap metabolic acidosis, with high lactate (3.3 mmol/L), that was resistant to IV fluid boluses. At that time, although his random blood glucose measures were ranging between 145 and 188 mg/dL, he had a blood pH of 7.225, with markedly decreased HCO_3_^−^ (14.60 mmol/L) and an anion gap of 17.40, consistent with moderate DKA. Urinalysis showed 3+ ketones (Table 2). Therefore, the decision was made to follow the DKA protocol for management in addition to closely monitoring his electrolytes and blood glucose levels. As soon as DKA was detected, he was started on IV fluids and insulin infusion. For fluids, he was given 1000 cc bolus of Ringer's Lactate followed by ½ Normal Saline (NS) at a rate of 150 cc/hour for one hour. Then, the patient was transitioned to Dextrose 5% ½ NS (150 cc/hour). After a few hours, the rate was decreased to 100 cc/hour. The next day, he was transitioned to 0.9% NS (70 cc/hour). The patient was kept on insulin for 12 hours from the time of EDKA diagnosis. Hours after treatment, the patient began to improve (Tables 1 and 2). His pH normalized, and his K+ level went down to 3.7 mmol/L. Urine ketones went from 3+ to 1+.

On the following day, the patient was awake and oriented. He did not have any active complaints. He was clinically and vitally stable. He received two units of packed RBCs due to his low hemoglobin (7.40 g/dL). His ABG normalized with a blood pH of 7.418, K+ of 3.90 mmol/L and an HCO_3_^−^ level of 23.3 mmol/L. At that time, his urinalysis was negative for ketone bodies. The next day, the patient was shifted to the cardiology floor with improving overall health status and hemoglobin level. He has had an uneventful hospital course for 8 days during which he was steadily improving. Subsequently, the patient was discharged home in a stable condition with appropriate follow-up appointments.

## 3. Discussion

The diagnosis of EDKA is challenging for several reasons. The fact that EDKA is rare, and more so in patients with T2DM [5], the nonspecific symptoms of the illness, and the absence of alarmingly high BG levels (i.e., >250) are all contributing factors that allow the diagnosis to be easily missed. The rarity of such an illness could be due to underdiagnosis, underreporting, and more likely, due to true rarity in occurrence. The recent possible role that SGLT-2i may play in the development of EDKA has encouraged physicians and researchers alike to uncover novel information regarding the incidence and underlying mechanism of the illness associated with these agents.

SGLT-2i are a new class of antidiabetic medications that lower BG levels by inhibiting glucose reabsorption in the renal tubules. It is suggested that SGLT-2i can lead to EDKA by decreasing BG levels, which leads to a reduction in insulin secretion and consequent rise in glucagon. In addition, SGLT-2i can directly stimulate glucagon production. As a result of low insulin and high glucagon levels, ketogenesis will be activated [5]. One study estimates a 7-fold increase in DKA in patients with T2DM due to SGLT-2 inhibitor use [6]. However, not all of these cases were euglycemic.

SGLT-2i have demonstrated cardioprotective effects and reduction in cardiovascular negative outcomes making them an excellent choice for patients with diabetes and CVD, such as our case [7]. The benefits of SGLT-2i in such patients must be outweighed against the risks of adverse effects, especially in times of stress (i.e., CABG), and the current recommendations suggest the omission of SGLT-2i dose one day before surgery [8]. Nevertheless, there have been cases described of perioperative EDKA in patients who have had these medications omitted for up to 48 hours [9]. In accordance with the current recommendations of the American Association of Clinical Endocrinologists and American College of Endocrinology [8], empagliflozin was omitted 24 hours before our patient's surgery, yet he still developed EDKA postoperatively. The time since the medication is held prior to surgery is an important factor to consider, due to two main reasons: the absence of the offending agent (i.e., gliflozin use) and the patient being put on insulin. Being on insulin for a greater period of time (e.g., >48 hours) is theoretically expected to decrease the chances of gliflozin-induced EDKA. The suggested hypothesis is related to low blood insulin levels and resultant switch of glucose to fat metabolism and subsequent development of ketosis and ketoacidosis. This is of important relevance in patients taking SGLT-2i, as they were most likely put on the medication due to its positive glycemic and cardioprotective effects [10]. Moreover, the fasting state prior to surgery presents its own added risk to developing EDKA, perhaps due to increased ketogenesis during periods of starvation, adding another factor that can possibly lead to, or exacerbate, this patient's condition [11].

In addition to highlighting the relationship between the offending agent (SGLT-2i) and the population at risk, it is worth focusing on the parts of the story that made the diagnosis challenging. In our case, the diagnosis of EDKA was not easy since the patient was sedated after CABG; thus, the common clinical presentation of DKA (abdominal pain, polyuria, excessive thirst, nausea, and vomiting) could not be appreciated, unlike other cases wherein patients were awake, oriented, and had typical symptoms of DKA [5, 12]. Additionally, his blood glucose levels postoperatively were well below 200 mg/dL; thus, it could have discouraged the caring physician to initiate continuous insulin infusion, which is the usual practice in most of the critically-ill or post-major surgery patients with diabetes. The ABG that showed anion gap metabolic acidosis that is resistant to IV fluids prompted further workup, wherein ketonuria was detected. Supplementary Figure 1 represents the connection between T2DM, SGLT-2I, and major stressors with EDKA development.

## 4. Review of the Literature

To better evaluate EDKA after CABG, we performed a literature search on PubMed (19 April 2021) using the following search string: ((Euglycemic Diabetic Ketoacidosis) AND (coronary artery bypass surgery)) without time restrictions, in addition to carefully assessing the references of certain EDKA articles for possible matches. Out of 67 PubMed articles, 5 articles were retrieved that reported on a total of 7 patients and were published between 2018 and 2021.  Table 3 summarizes the reported cases of EDKA after CABG in the literature. Interestingly, our search string did not include any keywords related to SGLT-2i; however, all the retrieved articles had SGLT-2i use in common. The time since gliflozin was held preoperatively was reported to be within 1 day prior to surgery in most of the cases; however, it was still possible for patients to develop EDKA when withholding the medication was carried out 42 and 48 hours before CABG [13, 16]. Moreover, most of the patients showed some signs and symptoms that usually accompany DKA, unlike our patient. Serum lactate was normal in the majority of cases, and even the patients who have had high serum lactate (e.g., 2.24, 2.8) did not reach to a clear cutoff level that is equivalent to lactic acidosis (serum lactate >5.0 mmol/L) [18]. Moreover, the time between CABG and EDKA development can extend up to a few days, as Zhand and Tamilia reported about their patient [15]. Of note, one article reported on a patient with EDKA after CABG who was on SGLT-2i that was held for >24 hours prior to surgery [19]; however, this article was not included in our table since it is in Japanese and, thus, could not be thoroughly analyzed.

Based on the current evidence, physicians must maintain a high index of suspicion for EDKA when dealing with cardiac patients on SGLT-2i, especially when these patients are to experience periods of excessive stress. This may be due to the absence of the easy and quick screening measure of typical hyperglycemia and the absence of clinical symptoms, especially in sedated patients. Patients with EDKA need immediate referral for emergency evaluation and management. The treatment includes addressing the underlying etiology, correction of dehydration with intravenous fluids, correction of electrolyte abnormalities, and the use of insulin and dextrose infusions until the anion gap and bicarbonate levels normalize [20]. The correct diagnosis in our patient was made mainly through laboratory investigations; prompt EDKA treatment protocol ensued that resulted in improvement of our patient's clinical status and outcome.

## 5. Conclusions

Patients with T2DM and CVD are recommended to be on SGLT-2i due to their positive CV benefits; however, these patients may also be considered having a higher risk for developing EDKA following an acute cardiac or other stressful event. Our case does not underestimate the importance of SGLT-2i use in cases where they are clearly indicated, but rather, it shows a potential side effect that can be avoided.

In conclusion, physicians should be aware and maintain a high index of suspicion for EDKA in patients taking SGLT-2i that have recently experienced high levels of stress (i.e., CABG). The diagnosis is challenging due to the rarity, nonspecific symptoms, and absence of high BG levels. Moreover, it is more challenging to make the diagnosis in sedated patients as the typical clinical presentation may be masked. Emergent treatment and multidisciplinary follow-up are needed to improve patient outcomes and mitigate complications. More studies on bigger scales are required to better understand the prevalence, pathophysiology, and management of this condition. Future guidelines should consider identifying the optimal time to discontinue SGLT-2i prior to a major surgery. In addition, we suggest routine urine ketone check in patients on SGLT-2i after CABG due to the seriousness of this condition, and the cost effectiveness of urine ketones makes them an excellent choice as a possible screening tool for such patients. An effort within the medical community must be put forth in future studies to merge and review similar cases to uncover more information that will help guide clinicians in EDKA prevention, detection, and treatment.

## Figures and Tables

**Table 1:** Blood tests of our patient at 5 different occasions.

Time^a^					
Labs (unit)	OPD (2 months)	Preop	Postop^*∗*^	Postop^	Postop day 1
RBS (mg/dL)	—	130–210 ↑	145–188	166	72–189
FBS (mg/dL)	146 ↑	—	—	—	—
HBA1c (%)	10.24 ↑	8.38 ↑	—	—	—
Na^+^ (mmol/L)	—	140	150 ↑	152 ↑	146
K^+^ (mmol/L)	—	4.34	4.37	4.60	3.55
Cl^−^ (mmol/L)	—	105	110 ↑	113 ↑	110 ↑
HCO_3_^−^ (mmol/L)	—	22	19 ↓	15 ↓	27
Creatinine (mg/dL)	1.47 ↑	1.11	1.03	1.19	1.18
BUN (mg/dL)	—	20	22 ↑	23 ↑	20
e-GFR (mL/min)	51 ↓	70	76	64	65
HGB (g/dL)	13.70	12.70	9.20 ↓	8.20 ↓	7.40 ↓
WBC (K/UL)	4.37 ↓	4.58	15.62 ↑	13.12 ↑	8.26
PLT (K/UL)	260	227	138 ↓	166	111 ↓

^a^Time represents the time in which the blood samples were taken, not the results. ^*∗*^Postoperative day 0 immediately after the patient was shifted to the ICU; hour 0. ^Postoperative day 0; hour 6. RBS: random blood glucose; FBS: fasting blood glucose; HBG: hemoglobin; WBC: white blood cells; PLT: platelets; OPD: outpatient department; preop: preoperatively; postop: postoperatively.

**Table 2:** ABG and urinalysis of our patient at 4 different occasions.

Time				
ABG (unit)	Preop	Intraop	Postop^	After DKA Tx
pH	—	7.398	7.225 ↓	7.403
pCO2 (mmHg)		42.30	34.30 ↓	40.10
HCO_3_^−^ (mmol/L)		25.40	14.60 ↓	24.70
Lactate (mmol/L)		1.4	3.3↑	1.3
Na^+^ (mmol/L)		148	147	146
K^+^ (mmol/L)		4.40	4.50 ↑	3.70
Cl^−^ (mmol/L)		110 ↑	115 ↑	113 ↑
Ca^2+^ (mmol/L)		1.00 ↓	1.10 ↓	1.08 ↓
Urine				
Glucose	4+ ↑	—	4+ ↑	4+ ↑
Protein	Negative		Negative	Negative
Ketone	Negative		3+ ↑	1+ ↑

^Postoperative day 0; hour 6. ABG: arterial blood gas; preop: preoperatively; intraop: intraoperatively; DKA: diabetic ketoacidosis; Tx: treatment.

**Table 3:** Reported cases of EDKA after CABG.

Author (year)	Age (years)	Gender	Comorb	Risk factor/s (mg)	Hours since gliflozin was withheld before CABG	Time between CABG and EDKA	Clinical presentation	BG (mg/dL)	pH	Blood ketones (mmol/L)	Anion gap (mmol/L)	Ketones in urine	Lactate (mmol/L)	Other T2DM medications
Lau et al. (2018) [13]	54	M	T2DM	Empagliflozin (25)	48	Hours	Nausea, vomiting, tachypnea	216	7.24	5.26 (b-HB)	15	N/A	1.1	NPH insulin
	58	M	T2DM	Empagliflozin (25)	28	Next day	Tachypnea	111.6	7.30	4.63 (b-HB)	14	Present	1.4	Metformin, gliclazide
	54	M	T2DM	Empagliflozin (25)	20	Next day	Nausea, tachypnea	172.8	7.33	0.98 (b-HB)	12	Present	2.8	Metformin

Chacko et al. (2018) [14]	66	F	T2DM, HTN, obesity, anxiety disorder	Dapagliflozin (N/A)	N/A	20 hours	N/A	165.6	7.26	1.8	N/A	N/A	1.13	Saxagliptin, metformin

Zhang et al. (2018) [15]	70	M	T2DM, PAF, DL	Empagliflozin (N/A)	N/A	Few days	Nausea, vomiting, weakness	201.6	7.27	>3.2 (b-HB)	31	Present	Normal	Metformin, liraglutide, modified release gliclazide

Osafehinti et al. (2021) [16]	60	M	T2DM, HC	Empagliflozin (10)	42	Hours	N/A	138	7.275	6.52 (b-HB)	25	N/A	Normal	Glimepiride, metformin, semaglutide

Pontes et al. (2021) [17]	57	M	T2DM	Dapagliflozin (10)	24	Next day	Tachypnea, restlessness, polydipsia, polyuria	151–248	7.21	N/A	30	Present	2.24	Basal insulin

Comorb: comorbidities; CABG: coronary artery bypass grafting; EDKA: euglycemic DKA; BG: blood glucose; T2DM: type 2 diabetes mellitus; b-HB: b-hydroxybutyrate; HTN: hypertension; PAF: paroxysmal atrial fibrillation; DL: dyslipidemia; HC: hypercholesterolemia; N/A: not available.

## Data Availability

The clinical data used in this case report are presented in this article. Literature review results and the search string used are fully described within the manuscript.

## Supplementary Materials

Supplementary Materials Supplementary Figure 1: the euglycemic diabetic ketoacidosis triad. EDKA: euglycemic diabetic ketoacidosis, T2DM: type 2 diabetes mellitus, SGLT-2I: sodium-glucose cotransporter-2 inhibitors.
