# Supplementary material for: Euglycemic Diabetic Ketoacidosis in a Sedated Patient after Coronary Artery Bypass Grafting: A Case Report and Literature Review
Source: Case Rep Med. 2021 Nov 18;2021:2086520. doi: 10.1155/2021/2086520 (PMC8616646; doi:10.1155/2021/2086520)
Supplement: Supplementary Materials — Supplementary Figure 1: the euglycemic diabetic ketoacidosis triad. EDKA: euglycemic diabetic ketoacidosis, T2DM: type 2 diabetes mellitus, SGLT-2I: sodium-glucose cotransporter-2 inhibitors. [file 2086520.f1.docx]

**Supplementary Figure 1:** The euglycemic diabetic ketoacidosis triad


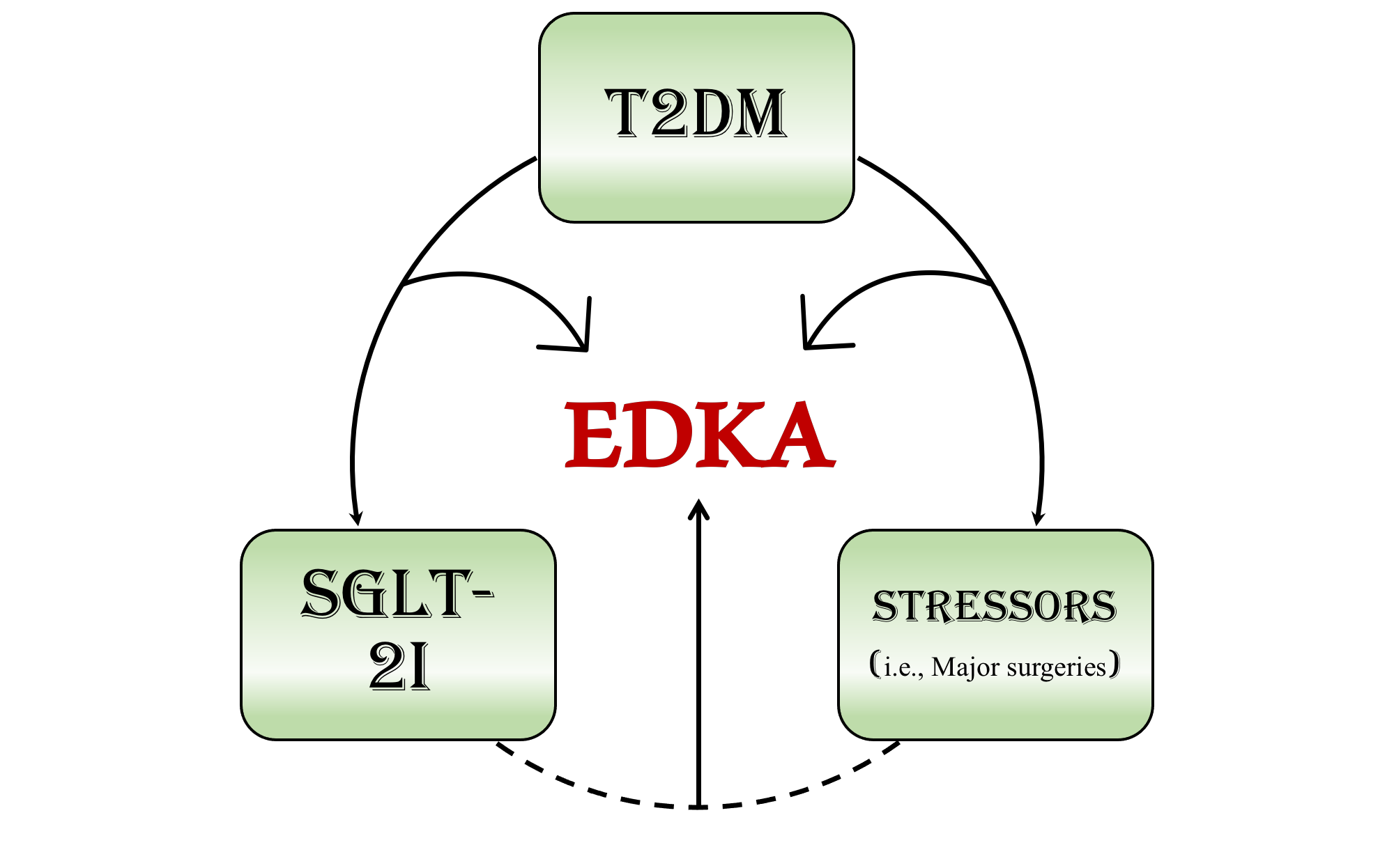


EDKA: Euglycemic diabetic ketoacidosis; T2DM: Type 2 diabetes mellitus; SGLT-2I: Sodium-glucose co-transporter-2 inhibitors
